# Distributed and Communication-Efficient Spatial Auto-Correlation Subsurface Imaging in Sensor Networks

**DOI:** 10.3390/s19112427

**Published:** 2019-05-28

**Authors:** Maria Valero, Fangyu Li, Jose Clemente, Wenzhan Song

**Affiliations:** Center for Cyber-Physical Systems, University of Georgia, Athens, GA 30602, USA; fangyu.li@uga.edu (F.L.); jclementes@uga.edu (J.C.); wsong@uga.edu (W.S.)

**Keywords:** sensor networks, communication-reduced, subsurface imaging, cross-correlation, spatial autocorrelation, ambient noise

## Abstract

A wireless seismic network can be effectively used as a tool for subsurface monitoring and imaging. By recording and analyzing ambient noise, a seismic network can image underground infrastructures and provide velocity variation information of the subsurface that can help to detect anomalies. By studying the variation in the noise cross-correlation function of the noise, it is possible to determine the subsurface seismic velocity and image underground infrastructures. Ambient noise imaging can be done in a decentralized fashion using Distributed Spatial Auto-Correlation (dSPAC). In dSPAC over sensor networks, the cross-correlation is the most intensive communication process since nodes need to communicate their data with neighbor nodes. In this paper, a new communication-reduced method for cross-correlation is presented to meet bandwidth and cost of communication constraints in networks while ambient noise imaging is performed using dSPAC method. By applying the proposed communication-reduced method, we show that energy and computational cost of the nodes is also preserved.

## 1. Introduction

Over the last years, ambient noise imaging, a well-known subsurface imaging approach, has become one of the fastest growing research areas in seismology and exploration geophysics. Compared to earthquake-based seismic tomography methods, ambient noise imaging is particularly useful in imaging shallow earth structures [1,2]. Moreover, because of the persistent nature of the seismic background noise, temporal variation of the earth structure can be studied and monitored by studying the variation in the noise cross-correlation function [3,4]. Ambient noise methods have the advantage of resistant repeating sources, low cost, and minimum environmental disturbance.

Seismic sensors are currently used for gathering seismic data that are later processed to obtain subsurface images using ambient noise methods [5]. Current approaches employ image reconstruction methods that rely on a centralized approach for processing the raw data captured by these seismic sensors. A solution can be the use of wired sensor communication approaches; however, the length limitation in cable communication represents a problem in large deployments. In addition, the centralized processing and computing style is not capable of being implemented in-situ and real-time subsurface imaging in all circumstances, especially in harsh environments [6]. It seems that a good solution for introducing in-situ and real-time imaging on sensors is wireless communication. For example, wirelessly connected sensors are deployed using an air-dropped way to monitor live volcano activities, where communication and computation become bottlenecks [7]. Recently, seismic tomography has been implemented using advanced wireless sensor networks with distributed computing algorithms [8,9,10]. The distributed style has advantages in reducing the data loss risk in the case of node and cable failures, because the sensing, computing, and data storage tasks can be operated in the sensor nodes. Instead of collecting data into a processing center, distributed seismic data processing and computing can be performed on individual sensors with communications among the local sensor array. Even though system-level challenges of deploying wireless sensor networks exist, focusing on distributed in-network signal processing and computation can help support real-time tomographic imaging.

**Figure 1:**
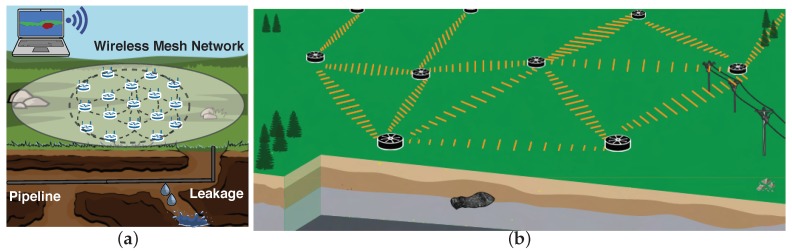
Concept of ambient noise seismic imaging on distributed sensor networks: (**a**) small array (m); and (**b**) large array (km).

We have been pioneers in developing such kind of systems [9,10,11]. Sensors are deployed in the field (Figure 1a is a illustrative example of deployment at meter scale, and Figure 1b at kilometers scale (the based method used in this paper (SPAC) has been tested in deployments in the range of few meters to several kilometers [12,13,14])) in a mesh network to work cooperatively and image the subsurface. Every node gathers independently ambient noise raw data, computes in-situ signal preparation [15], communicates with immediate neighbors to share its narrow-data recording, performs cross-correlation with the signals that receives from its neighbors [1], applies spatial auto-correlation methodology [11] to estimate subsurface velocity, and talks again with neighbors to aggregate the final velocity maps that illustrate subsurface wave-speed variations [16]. A friendly user-interface helps scientists to visualize real-time seismic images and interprets results. For example, velocity variations in the final velocity map may help to locate underground pipelines and water leakages, as shown in Figure 1.

In distributed ambient noise imaging, the most intensive communication process is the transfer of data for cross-correlation [10]. To cross-correlate data, every node has to send its own data to the neighbor nodes. Even though some communication-reduction techniques can be applied (reduction of data using a pre-specific narrow band of frequencies, compression techniques, etc.), sending data to all neighbors can be inefficient. Furthermore, some nodes waste computational performance doing cross-correlations that can be done in other nodes.

In this paper, besides utilizing distributed and in-network computing to imaging shallow subsurface, we propose a new communication-reduced method that can be applied in ambient noise imaging based on Distributed Spatial Auto-Correlation (dSPAC). The method is designed as a combinatorial optimization problem that first transforms the topology of the mesh network in a suitable graph for a transportation problem [17]. Then, the optimization problem is solved to get the best nodes for computing cross-correlation to meet network limitations. Constraints regarding bandwidth are added. Furthermore, constraints regarding the energy of the sensors can also be added to make a more energy-efficient selection. By applying the proposed communication-reduced method, we show that energy and computational cost of the nodes is also preserved. We show in our experiments that it is possible to image buried pipelines using our method, and potentially detect water leakage.

The main contributions of this paper can be summarized as:

- The in-situ and real-time computing of shallow subsurface imaging using a proposed distributed spatial autocorrelation technique and ambient noise is introduced.
- A novel communication-reduced method for neighborhood communication between nodes that allows them to estimate the correlation between signals of neighbor nodes using less energy and meeting bandwidth constraints is proposed.
- A field deployment illustrates the usability of the method for detecting shallow infrastructures. The small array was selected only for validation purposes. We emphasize that the same methodology can be used in large arrays since wireless communication can reach several meters, even kilometers with gain antennas.
- An analysis of bandwidth, energy, and communication cost of our method compared to other centralized approaches that require all data to be sent to a central unit is presented.

The rest of the paper is organized as follows: Section 2 provides an overview of distributed ambient noise imaging based on dSPAC and highlights the limitation of this approach without a communication-efficient method. Section 3 introduces the proposed communication-reduced model for dSPAC and algorithm. Experimental results and a deep analysis regarding communication, computation, and energy cost are conducted in Section 4. We present a real deployment experiment for studying ambient noise with dSPAC in Section 5. A discussion regarding robustness and communication limitation of the proposed approach as well as a comparison with the centralized approach is presented in Section 6. Finally, future work and conclusions are presented in Section 7 and Section 8, respectively.

## 2. In-Situ Cross-Correlation and dSPAC for Ambient Noise Imaging

**Figure 2:**
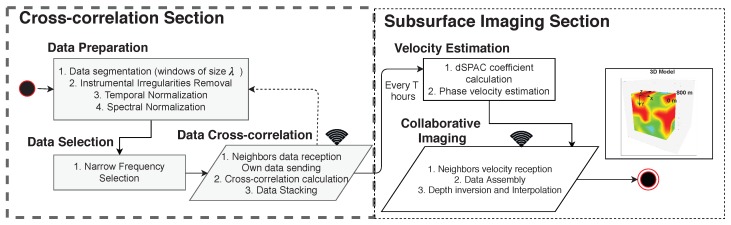
System methodology for distributed Spatial Autocorrelation (dSPAC) subsurface imaging.

The work-flow of in-situ cross-correlation and dSPAC for estimating ambient noise imaging is described in Figure 2. The process consists of two main sections: cross-correlation and subsurface imaging. The main idea of the methodology is every sensor cross-correlate its data with the data of its neighbors in a time window λ. To do so, nodes broadcast their data. However, instead of broadcasting their raw data, nodes broadcast prepared and selected data to diminish the communication cost. The cross-correlation process is continuous. Once a time *T* is complete, nodes perform the subsurface imaging by estimating the velocity variation using dSPAC method. Then, a collaborative image is produced by aggregating in a collaborative and distributed way the velocity estimation of each node. In this section, we introduce the complete overflow by presenting the mathematical framework and algorithm that includes each step. Then, we discuss why, even though the method produces promising results, the communication cost needs to be improved.

### 2.1. System Model

Each sensor is provided with a radio system to communicate with the rest of the network, but each radio system has only a limited range for transmissions and receptions. It is assumed that the transmission range and the reception range are the same, and it is referred to as communication range. Consequently, each node is able to communicate with a restricted number of other sensors, i.e., the ones deployed within its communication range. In this work, we use an undirected graph G=(V,E) to model the topology of the network. Each node in V represents a sensor node, and the link (i,j)∈E represents that nodes *i* and *j* are in communication range and they are considered as neighbors. Let us denote with N(i) the set of neighbors of node *i*. Let |V| denote the number of nodes in the network.

Let $x_{i}\left(t\right)$ be the raw signal of sensor node *i* in the time *t*. Every node in V gathers $x_{i}\left(t\right)$ and starts the preparation for cross-correlation. Here, x is a vector that contains the readings of ambient noise for a time λ; e.g., λ can be equal to 1 min, 2 min, 5 min, etc. (Note that, because we are doing continuous monitoring, we chose non-overlapping time, following the authors of [15,18,19]. If the seismic survey is time-limited, only 1 or 2 h, we can use overlapping to improve the convergence of the cross-correlations.)

### 2.2. Signals Cross-Correlation

After gathering $x_{i}\left(t\right)$, every sensor performs a *data preparation*. The purpose of this preparation is to accentuate ambient noise by attempting to remove earthquake signals and instrumental irregularities that tend to hide the ambient noise [15]. To *remove instrumental irregularities*, we withdraw the mean and the trend of the signal [20]. Then, we apply running-absolute-mean method [15] for *temporal normalization*. For the raw data $x_{i}\left(t\right)$, the normalization weight is

(1) $$\begin{array}{cc} W\left(t\right)=\frac{1}{2q+1}\sum_{j=-q}^{q}x_{i}\left(m-j\right) & \;\mathrm{for}\;m=q+1,q+2,\ldots ,t-q \end{array}$$

and the normalized data are $\hat{x_{i}}\left(t\right)=x_{i}\left(t\right)/W\left(t\right)$. The width of the normalization window is 2q+1. Finally, *spectral normalization* [15] is applied to reduce broad imbalances in single-station spectra to aid in the production of a broad-band dispersion.

Once data have been prepared, a *data selection* process is conducted. In the data selection, a narrowed band-pass filter is applied to keep only a range of frequency components we need to study. This process is known as *narrow frequency selection*. Let

$$\Psi =\left\{f_{1},\ldots ,f_{m}\right\}\subset \left\{0,1,\ldots ,N-1\right\}$$

denote the indices of the narrowed frequency components, which is the same across all sensors. Sensor *i* only transmits the subset of frequency samples $\left\{X_{i}\left(f_{k}\right)\right\}$, where $f_{k}\in \Psi$. Assuming that m=|Ψ| frequency samples are selected; then, the amount of data to be transmitted is reduced from O(N) to O(m). In this case, N≫m because we have to observe long enough noise sequence and the frequency band we are interested is usually narrow. Most importantly, if the time *t* of stacking cross-correlation is not large (for example 1–10 min), and the frequency band is narrow, for example, 80–100 Hz (refer to Section 5.4 for details of frequency selection), we are able to achieve 80–90% reduction, and the data to transmit can be sent in only one UDP or TCP packet. This achievement significantly reduces the total communication overhead.

Node *i* then broadcasts $X_{i}\left(f_{k}\right)$ to N(i). Note that, at the same time, node *i* receives data that come from every node that belongs to N(i). For each node j∈N(i), node *i* computes

(2) $$C_{{\hat{x}}_{i}{\hat{x}}_{j}}={\hat{x}}_{i}⊗{\hat{x}}_{j}\equiv \frac{1}{2T}\int_{-T}^{T}{\hat{x}}_{i}\left(\tau \right){\hat{x}}_{j}\left(\tau +t\right)d\tau =X_{i}\left(f_{k}\right)\cdot \bar{X_{j}\left(f_{k}\right)}$$

where *T* is the total time of cross-correlation and $\bar{X}$ indicates the complex conjugate of X. The cross-correlation is $C_{{\tilde{x}}_{i}{\tilde{x}}_{j}}$, which is stacked with itself every time nodes send and receive data from their neighbors. The stacking process is usually employed to increase the signal-to-noise ratio (SNR) of the signal [21]. In this case, we stack the cross-correlation results every time it is performed every λ min, where λ can be 1, 2 or 5 min depending on the system configuration, until completing *T* time. For instance, if λ is 5 min, the node correlate the data and then stack them with the previous stacked 5 min and so on.

We use the symmetric component of the cross-correlation that is the average of the cross-correlation at positive and negative lags [15]. Thus, note that $C_{{\tilde{x}}_{i}{\tilde{x}}_{j}}=C_{{\tilde{x}}_{j}{\tilde{x}}_{i}}$. In addition, note that, since nodes broadcast to all their neighbors, the cross-correlation $C_{{\tilde{x}}_{i}{\tilde{x}}_{j}}$ is calculated in both node *i* and node *j*, which implies a wasting of computation. When the system has complete *T* time that involves multiple cross-correlations and stacking processes, the subsurface imaging section begins the distributed calculation of the velocity variation structure.

### 2.3. Subsurface Imaging

**Figure 3:**
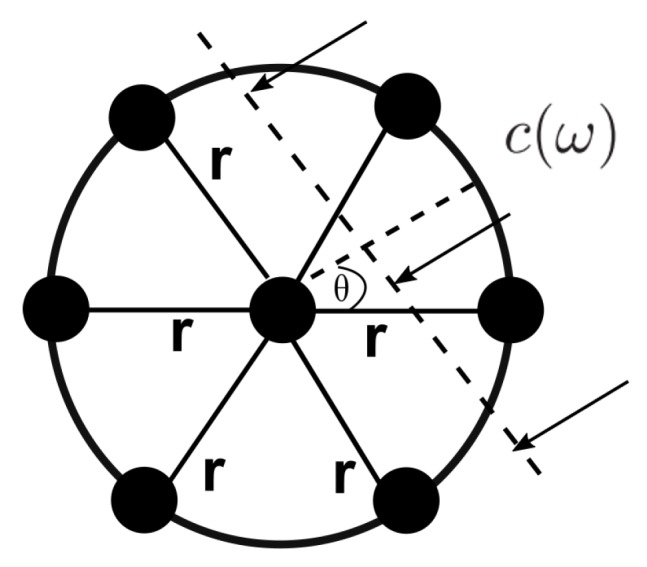
Geometry of sensor nodes and an incident plane wave. Filled black circles represent the sensors.

After cross-correlation, every node in V estimates locally the SPAC coefficients [22]. The SPAC method has exhibited a good performance in heterogeneous and isotropic media [23], and has shown a comparable output with other tomography methods such as interferometry [24]. Even if we use high frequencies study, the SPAC method holds even for a non-isotropic wavefield, because the normalized cross-spectrum can be averaged with respect to various incident directions by using the wavefield at the center of a circle and the wavefields on a circumference of the circle [24]. We called the method dSPAC because it is the distributed version of SPAC. The dSPAC method can extract the phase velocities of surface waves from microtremor array observations. The basic theory of the spatial auto-correlation method [23] is summarized as follows. Having an array of sensors (called receivers) equally spaced on a circle of radius *r* and having an extra receiver at the center, as shown in Figure 3, the phase velocities (c(ω)) can be calculated.

If microtremors are observed, the complex coherencies COH between a central and a circumferential receiver can be defined as:

(3) COH(r,ω,θ,ϕ)=exp{irkcos(ω−ϕ)},

where *i* is the imaginary number, ω is the angular frequency, *k* is the wavenumber, θ is the azimuthal angle and ϕ is the azimuth propagation of a single plane wave across the array. The dSPAC coefficients, also called azimuthal average, is defined then by:

(4) $$\rho \left(r,\omega \right)=\frac{1}{2\pi }\int_{0}^{2\pi }\exp\left\{irkcos\left(\theta -\phi \right)\right\}d\theta =J_{0}\left[\frac{\omega }{c(\omega )}r\right],$$

where $J_{0}$ is the Bessel function of the first kind of zero order. Here, *r* must be fixed. Because of the cos(ω−ϕ) symmetry in Equation (4), we can switch ω with ϕ and obtain the same result. This means that dSPAC coefficient can be estimated as the average of the cross-correlation between every node pair in a fixed geometry with the same ratio *r*, which remedies the biases in phase velocity measurements caused by a non-isotropic or directional wavefield. In other words, Equation (4) can be rewritten as:

(5) $$\rho_{i}\left(r,\omega \right)=\frac{1}{\left|N(i)\right|}\sum_{j=1}^{\left|N(i)\right|}C_{{\tilde{x}}_{i}{\tilde{x}}_{j}}\equiv J_{0}\left[\frac{\omega }{c_{i}\left(\omega \right)}r\right]$$

where *i* is the central sensor of the circular array. The phase velocities are estimated by fitting the observed dSPAC coefficients to the Bessel function. Note that a larger array in a circular topology can have multiple circular sink nodes, as shown in Figure 1a.

After the velocities $c_{i}\left(\omega \right)$ are estimated, the sink nodes at each ring broadcast the velocity information to the other sink nodes, and they perform an interpolation process to form a 3D map of the subsurface with all frequencies in consideration. Each layer of the 3D map represents a subsurface depth. With this information, we can analyze the velocity variations and determine the presence of structures, such as pipelines, within the subsurface.

### 2.4. Limitations of Broadcasting to All Neighbor Nodes

The main limitation of distributed subsurface imaging [10] is the broadcasting to all neighbors without distinguishing between them, and the multiple and needless computation of the same cross-correlation in different nodes. If $C_{{\tilde{x}}_{i}{\tilde{x}}_{j}}=C_{{\tilde{x}}_{j}{\tilde{x}}_{i}}$, it should be convenient to process the cross-correlation only one time. Furthermore, if we propose a mechanism to select the best nodes to compute cross-correlation meeting the bandwidth and communication constraints, some nodes can help others by computing cross-correlation between neighbor nodes too. In the next section, we introduce the proposed communication-reduced model for subsurface imaging using dSPAC to get the best nodes for computing cross-correlation to meet network limitations.

### 2.5. Scope of the Proposed Model

The proposed communication-reduced method focuses the attention in the cross-correlation section. We aim to reduce the number of cross-correlation and select the best nodes to compute them. Our results and analysis are based on the performance of the distributed system during this correlation section. The subsurface imaging section results are discussed in the Section 5. The idea is to present a model that can improve the communication cost and bandwidth utilization in the first part of the ambient noise process, which is the most communication intensive. In the next section, we detail the proposed model and define the main mathematical framework of the solution.

## 3. Communication-Reduced Model for dSPAC

In this section, we present the communication-reduced model for dSPAC in ambient noise imaging. Specifically, we improve the communication cost and bandwidth utilization in the *correlation section* of the system, where the communication for cross-correlation and stacking is the most intensive process. First, we explain why, even though a reduction of the data is performed in dSPAC methodology, the communication is still inefficient. Furthermore, the computation cost is high on all nodes in the network. Later, we present the model for selecting the best nodes to compute cross-correlation, and the optimization model to guarantee an appropriate solution.

**Figure 4:**
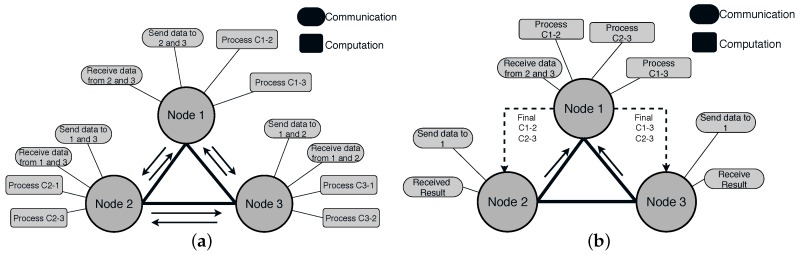
(**a**) Current communication pattern; and (**b**) proposed communication-reduced method example.

The naive communication pattern (Figure 4a) may include each sensor broadcasts its narrowed-data to its neighbors, and each one of them performs cross-correlation. However, after stacking cross-correlation, the result is symmetric [1]. This means the cross-correlation coefficients will be the same from Sensor 1 to Sensor 2, and vice-versa.

By taking advantages of symmetric cross-correlation [25], we can assume that one node (*A*) may compute the cross-correlation of its neighbor (*B*), and then forward the results. That means *A* does not send narrowed-data, only receives from *B* and computes $C_{AB}$. Furthermore, if *K* is neighbor of *A* and *B*, Sensor *A* may also compute the $C_{BK}$ among *B* and *K*. Figure 4a shows the current communication and computation pattern to perform cross-correlation. Each sensor is represented as an independent “node”. Every *t* time (*t* could be equal to 5 min, for example), nodes broadcast their data to their neighbors. For example, Node 1 receives data from Nodes 2 and 3 every 5 min. Node 1 computes the cross-correlation between itself and Nodes 2 and 3. The process is the same in Node 2 and 3. Notice that Node 2, for example, also computes Cross-Correlationa 1 and 2. This is also a waste of computation. If only Node 1 receives and computes, the communication cost and computation cost would be reduced significantly.

The proposed method for solving this problem is illustrated in Figure 4b, where Nodes 2 and 3 send data only to Node 1. Furthermore, to compute the cross-correlation between Nodes 2 and 3, there is no need for communication between those nodes because Node 1 can compute this cross-correlation too. The number of packets sent over the network is significantly reduced. Node 1 continues receiving data from Nodes 2 and 3 and stacking the cross-correlations until completing time *T*, which is the time to begin the subsurface imaging section. At that moment, Node 1 forwards back the results to Nodes 2 and 3.

However, in larger mesh networks, with more complex topology, finding the solution is not straightforward. An optimization scheme has to be formulated to solve the problem in the most efficient way. We designed a new communication-reduced method for cross-correlation. The method is designed as a combinatorial optimization problem that first transforms the topology of the mesh network in a suitable graph for transportation problem. Then, the optimization problem is solved to get the best nodes for computing cross-correlation. Constraints regarding bandwidth are added. Furthermore, constraints regarding the energy of the sensors can also be added to make an energy-efficient selection.

### 3.1. Problem Definition

**Figure 5:**
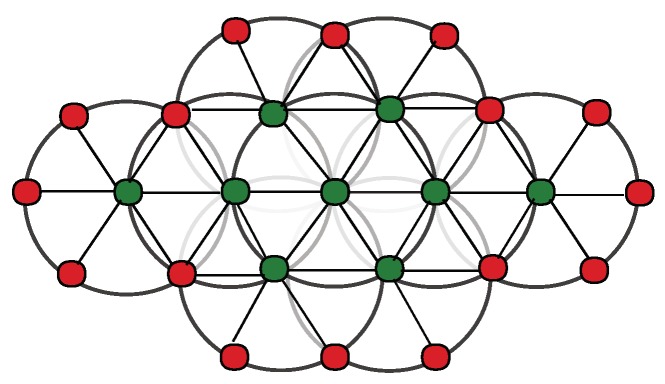
dSPAC topology example. Green circles are sink nodes and red circles are leaf nodes.

In our model, we have a mesh network of sensors represented as a weighted graph G=(V,E,λ), where each edge (u,v)∈E has a transmission cost $\lambda_{uv}$. The network G is assumed to have a suitable topology for dSPAC computation. Figure 5 shows an extended topology example that can be deployed for dSPAC-based ambient noise imaging. Note that other kinds of topologies, such as hexagons or triangles, can also be used. Nodes can only communicate directly with their direct neighbors, and the data they send should be computed at most in one hop of distance. We consider the data that each node sends during the *correlation section*.

**Assumption 1** (Fixed Packet Size)**.** *We assume without loss of generality that the size of the packets generated at the data producers have the same size and we consider each packet as a data element.*

### 3.2. Network Transformation

**Figure 6:**
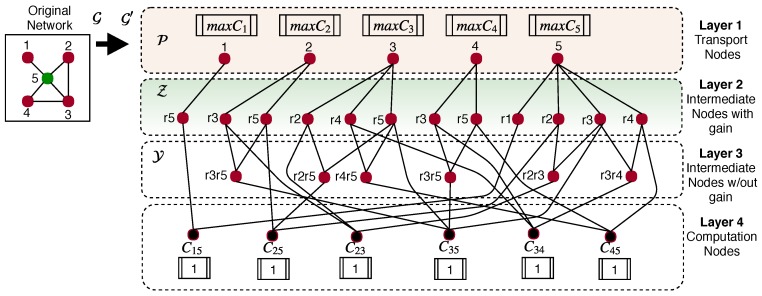
Network transformation example.

Before we can find the optimal solution to our problem, we need to transform our original network graph G into a flow network $G^{\prime }$. We propose using neighborhood information for this network transformation. A series of steps have to be performed to transform $G\to G^{\prime }$:

- We set up every node in V as a *transport node*. The set of transport nodes is represented as P (Figure 6, Layer 1). The transport nodes are considered to be the nodes that may compute the cross-correlations, and they can be constrained with the maximum number of cross-correlations to compute on it ($maxC_{i}$ where i∈P). If we assume that the computational cost is unimportant, then $maxC_{i}$ can be infinite. However, that is not the case for many nodes due to energy consumption. *Every transport node produces a cost of λ for every unit that sends to the second layer of nodes*. By default, nodes in P send one unit to Layer 2.
- We set up a set of *intermediate nodes with gain* Z as the Layer 2 of the flow network (Figure 6, Layer 2). This layer is composed for the neighbors of each node i∈P. For example, if in the original network, Node 2 (i=2 and i∈P) has two neighbors (Nodes 3 and 5), then two new nodes will be added to Layer 2 (Z), called r3 and r5, which are going to be directly connected to Node 2 in Layer 1 (P). Every node in Z receives one unit from the transport nodes, and it generates half unit (0.5) for each connection with the Layer 3, or they generate 0 units if they do not have neighbors. There is no cost of transporting data from Layer 2 to other layers.
- We set up the Layer 3 as the set of *intermediate nodes without gain* Y. This layer of nodes (Figure 6, Layer 3) is composed by the neighbors of node j∈Z in Layer 2 that also are neighbors of i∈P in Layer 1. This layer is used to analyze the neighbors that are able to compute the cross-correlation of other neighbors but not more than one hop of difference. For instance, Node r3 from Z that is connected to Node 2 from P is, itself, neighbor of Node 5 in the original network. Node 2 is also a neighbor of Node 5 in the original network. Then, we add Node r3r5 to the Layer 3 because Node 3 is neighbor of Node 5 and both are neighbors of Node 2 in the original network. This layer does not generate any cost for unit.
- The Layer 4 is composed of all the possible cross-correlations between neighbors the system needs to compute. For example, in the original network of Figure 6, we need to compute the cross-correlations $C_{1-5}$, $C_{2-5}$, $C_{2-3}$, $C_{3-5}$, $C_{3-4}$, and $C_{4-5}$ because those are the neighbors (there is exists a edge) of the nodes in the network.

After the node transformation, we can set the optimization problem over $G^{\prime }$ by minimizing the communication cost in that network. An important comment regarding Layer 2 is that, as mentioned, those nodes generate 1/2 or 0.5 unit for each connection to Layer 3. To clarify this issue, consider the following example: In Figure 6, Node 2 (Layer 1) is connected to Nodes r3 and r5 in Layer 2 and transfer one unit at a cost λ. Then, Node r3 generates 1/2 unit and send it to Node r3r5 (Layer 3); similarly, Node r5 generates 1/2 unit and send it to Node r3r5 too. This makes a total of 1 unit to Node r3r5 and guarantees that the optimization problem can study this possible solution.

After graph transformation, we can set up the optimization problem for minimizing the communication cost between the nodes by selecting the best nodes to compute cross-correlations.

### 3.3. Model Design

We want to minimize the cost of transmission (λ) of the number of packets (β) requires cross-correlation. The idea is to perform all the needed cross-correlation with the minimum cost of transmission. Let $\lambda_{uv}$ be the cost of transmission from node *u* to node *v*, and $\beta_{uv}$ the number of packets to transmit from node *u* to node *v*. The maximum number of packets to transmit simultaneously by a node *u* is defined by $\chi_{u}=\Omega_{u}/S_{u}$ where $\Omega_{u}$ is the available bandwidth in node *u* and $S_{u}$ is the size of the packet.

Table 1 summarizes the main variables of our optimization problem. We define the optimization problem as:

(6a) $$\begin{array}{c} \mathrm{minimize}\;\sum_{uv\in E}\lambda_{uv}\beta_{uv} \end{array}$$

(6b) $$\begin{array}{ccc} s.t:\\ - & \sum_{l\in \Gamma_{u}}\beta_{ul}\leq \chi_{u} & \end{array}$$

(6c) $$\begin{array}{c} \sum_{k\in \Upsilon_{u}}h\beta_{ku}-\sum_{l\in \Gamma_{u}}\beta_{ul}=0 \end{array}$$

(6d) $$\begin{array}{c} \sum_{k\in \Upsilon_{u}}\beta_{ku}-\sum_{l\in \Gamma_{u}}\beta_{ul}\geq 0 \end{array}$$

(6e) $$\begin{array}{c} \beta_{ku}=\frac{1}{2}\forall k\in r,u\in g\\ \sum_{k\in \Upsilon_{u}}\beta_{ku}=1 \end{array}$$

The objective function (6a) is to minimize the communication cost between nodes in the network to meet bandwidth specifications. The constraint in Equation (6b) is established for Layer 1 (*transport nodes*), and it guarantees that the number of packets to be transmitted will be less or equal to the maximum number of packets to transmit simultaneously in the edge. Note that *l* in $\beta_{ul}$ belongs to the set of nodes connected by output edges with node *u* where *u* is the node in consideration in Layer 1. The constraint in Equation (6c) is established for Layer 2 (*Intermediate nodes with gain*). This constraint guarantees that the inflow packets will be the same than the outflow packets, but it will be a gain of *h*, where *h* is 1/2 for each connection to Layer 3, and it is calculated using:

(7) $$h=1+\frac{\left|N(k)\cap N(u)\right|}{2}.$$

Note that N(k) is set of neighbors of *k*, and N(u) is the set of neighbors of *u*; consequently, |N(k)∩N(u)| represents the number of neighbors of *k* that also are neighbors of *u*.

The constraint in Equation (6d) is established for Layer 3 (*Intermediate nodes without gain*). This constraint guarantees that the inflow packets will be equal or greater than outflow packets in this layer. The inflow is equal to the inflow when the node in Layer 3 receives packets from its two connection with Layer 2 (that means that the cross-correlation can be done); otherwise, the outflow is 0. Note that, in this case, for all intermediate nodes without gain, the input should be 1/2 for all k∈r, where *r* is the set of intermediate nodes with gain, and u∈g where *g* is the set of intermediate nodes without gain. Finally, the constraint in Equation (6e) is established for Layer 4 (*computation nodes*). This is equal to 1 because we want to compute only one cross-correlation per pair.

## 4. Experiments and Evaluation

We conducted a series of experiments to test our communication-reduced model. In this section, we explain the main results and improvements in terms of bandwidth, energy consumption, and computational cost.

### 4.1. Topology Design

**Figure 7:**
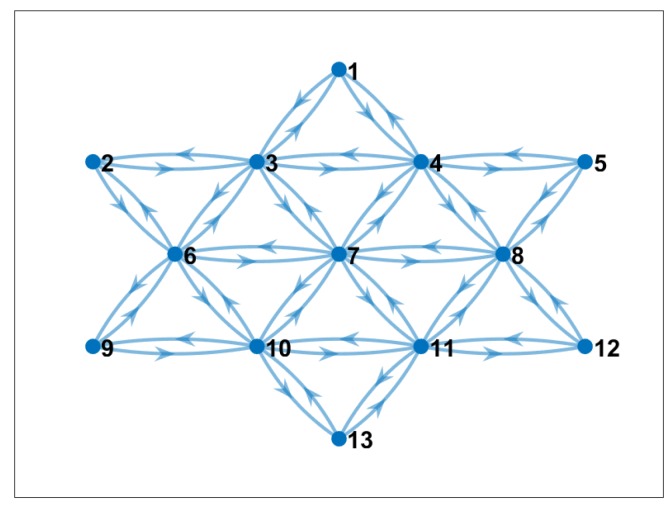
Communication pattern without the proposed communication-reduced method. Nodes broadcast data to their neighbors.

Because dSPAC-based method requires a ring topology, we used this kind of arrangement in our experiment. We show that this topology is also suitable for real-world experiments below. The used topology is shown in Figure 7.

To execute the dSPAC method with this specific topology, the total number of cross-correlation that needs to be calculated is 24. This number is based on the number of neighbors that are formed in the mesh network. The list of needed cross-correlations is shown in Table 2. For the sake of space, we do not show the graph transformation of the topology in Figure 7.

### 4.2. Experiment 1: Unlimited Bandwidth

**Figure 8:**
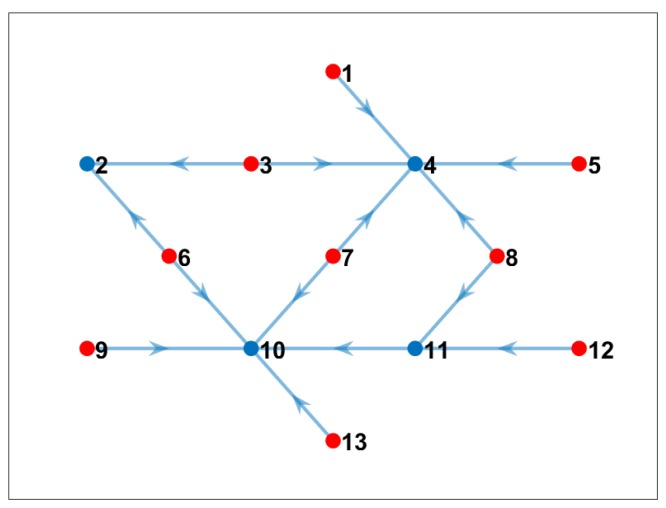
Communication pattern using the proposed model when we considered unlimited bandwidth (blue nodes are nodes that computed cross-correlation; red nodes only sent data to the corresponding network).

If we assume all nodes have unlimited bandwidth, and they can send and receive from/to any neighbor node, the proposed communication-reduced model estimates the optimal solution such as the one shown in Figure 8. Note that all 24 cross-correlations were calculated in the “best node” and the communication cost was minimum for this scenario. In the figure, red nodes were selected to be just sender of data, and blue nodes are nodes that computed cross-correlations. Note also in Table 3 that only five nodes used computational resources. However, some nodes, such as Nodes 8 and 10, compute more than seven or eight cross-correlations from its neighbor nodes, which may imply a bottleneck and affect the energy consumption of those nodes. The advantage of this solution is the main sink node (Node 7), which is the one that computes the 3D interpolation of the subsurface image, do not waste computation time and energy during the cross-correlation process.

To further measure the bandwidth constraint in the performance of the proposed model, we performed two more experiments by limiting the number of neighbor nodes data that a node can receive to compute cross-correlations. We present the results in Experiments 2 and 3 below.

### 4.3. Experiment 2: Limited Bandwidth

We performed two different tests with limited bandwidth considering that one node only can receive data from up to: (i) four neighbor nodes; and (ii) two neighbor nodes.

#### 4.3.1. Maximum Four Neighbors

**Figure 9:**
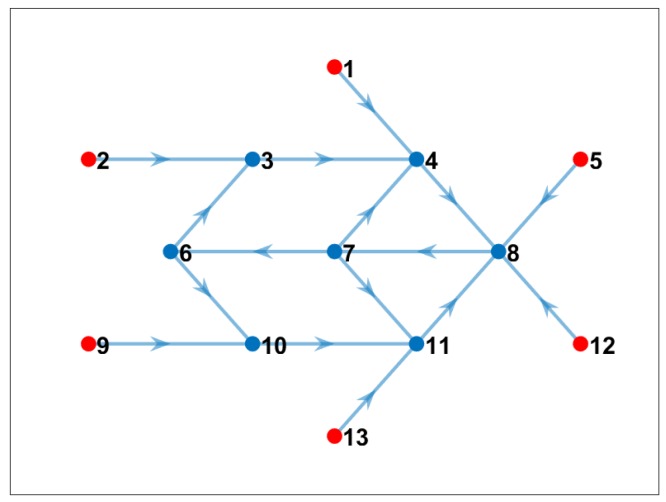
Communication pattern using the proposed model when we considered that one node only can receive data from up to four neighbor nodes (blue nodes are nodes that computed cross-correlation; red nodes only sent data to the corresponding network). Nodes that computed cross-correlations.

We conducted an experiment by considering that one node could only receive data from up to four neighbor nodes to compute cross-correlation. Figure 9 shows the optimal solution when we added this new constraint. Note that, in this case, the communication cost continued being the minimum possible under the constraints, and the computational cost was more evenly balanced between the nodes in the network, as shown in Table 4.

#### 4.3.2. Maximum Two Neighbors

**Figure 10:**
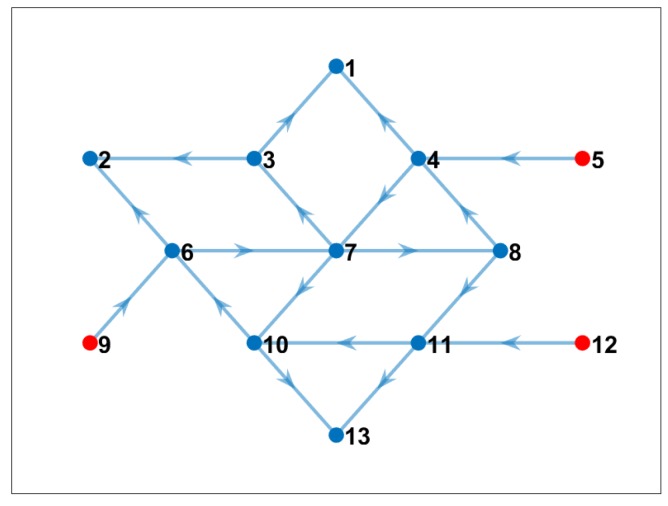
Communication pattern using the proposed model when we considered that one node could only receive data from up to two neighbor nodes (blue nodes are nodes that computed cross-correlation; red nodes only sent data to the corresponding network). Nodes that computed cross-correlations.

We also conducted another experiment by increasing the bandwidth limitations. We considered that one node could only receive data from up to two neighbor nodes to compute cross-correlation. Figure 10 shows the optimal communication pattern for this scenario. Note that the number of nodes that computed cross-correlations increased with this bandwidth limitation. As shown in Table 5, we could confirm that, for this specific topology and cross-correlation requirements, the maximum number of cross-correlation that a single node needed to compute was four. Note also that the computational cost for sink nodes was relatively low.

### 4.4. Experiment 3: Variable Bandwidth

In real scenarios, the bandwidth is variable and depends, among other things, on the number of connections in the topology. In a mesh network, every “*hop*” (link) between sensors will decrease the bandwidth by half [26]. This happens because wireless links can only do one thing at a time—transmit or receive. In a long “chain” of mesh links, this results in a very slow connection from end to end. Even though this estimation (half of the bandwidth decreasing by every link) is widely accepted, in reality, other factors can impact the available bandwidth in a specific time, for example communication range, other networks interference, etc.

As explained below, our real-world sensors are based on a Raspberry Pi 3 as computer board. The wireless communication bandwidth of Raspberry Pi 3 is estimated at ∼10 Mbps (Megabytes per second) [27]. Due to the number of links in our topology (some nodes may have five or six links, which reduced the available bandwidth), we based our observations on a maximum available bandwidth of ∼2 Mbps.

**Figure 11:**
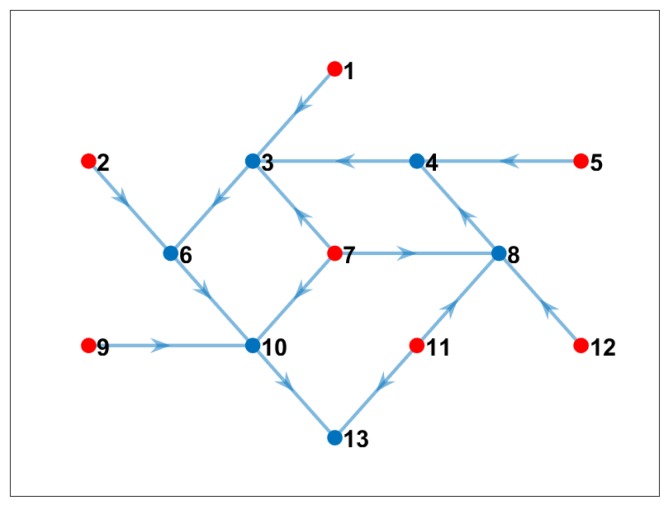
Communication pattern using the proposed model when we considered variable bandwidth (blue nodes are nodes that computed cross-correlation; red nodes only sent data to the corresponding network). Nodes that computed cross-correlations.

We set an experiment in which we varied randomly the available bandwidth between each pair of nodes depending on the number of links or hops, and we ran our optimization method. The “best nodes” to compute cross-correlations and the communication pattern is shown in Figure 11. Note that the central node, which had more neighbor connections, was not selected as one of the best nodes to compute. Table 6 shows that only six nodes computed all needed cross-correlations. We can conclude that it is possible to apply our communication-reduced method on nodes where variable bandwidth is present.

### 4.5. Bandwidth and Energy Analysis

To further analyze the results of the proposed method, we compared the original communication pattern (Figure 7) with the results of our experiments using the proposed communication-reduced method. We analyzed three main aspects: (i) throughput of the network; (ii) computational cost in terms of the number of computed cross-correlations; and (iii) percentage of energy saving in the network after applying the proposed method.

**Figure 12:**
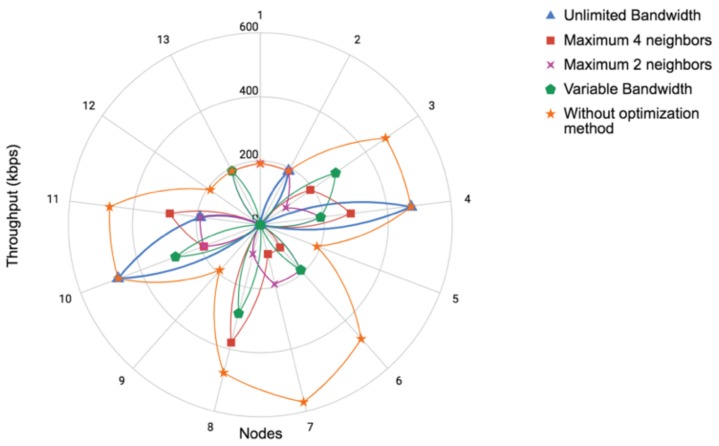
Throughput comparison of every sensor node among different available bandwidth using our proposed communication-reduced method vs. communication pattern without communication-reduced method. Note that our method improved bandwidth utilization in all the tested scenarios.

**Figure 13:**
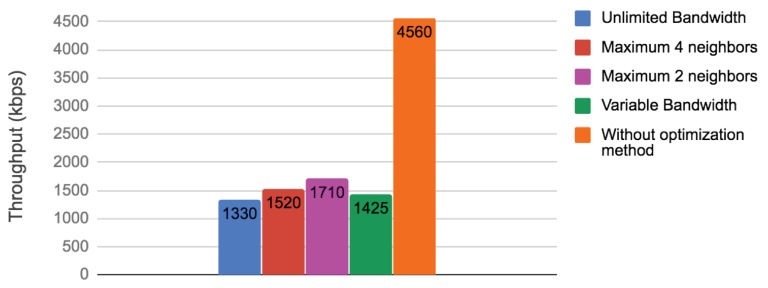
Total throughput comparison node among different available bandwidth using our proposed communication-reduced method vs. communication pattern without communication-reduced method. Note that in the whole system level our method met bandwidth constraints.

We measured the throughput on every sensor node based on the number of packets received to verify if our method improved bandwidth utilization. Figure 12 shows for each node the number of kilobytes per second (kbps) transmitted. Note that the original communication pattern (without optimization method) utilized much more bandwidth than our method with or without bandwidth restriction. Only the unlimited bandwidth experiment had two nodes that utilized similar bandwidth to the original pattern. Figure 13 shows the total throughput of the network in all cases. Our method significantly improved the communication cost. Between our experiments, Experiment 1 (assuming unlimited bandwidth) reduced communication the most. However, in reality, we did not have unlimited bandwidth, and the option of our four experiments (variable bandwidth that depends on the number of links/hops) resulted more attractive.

**Figure 14:**
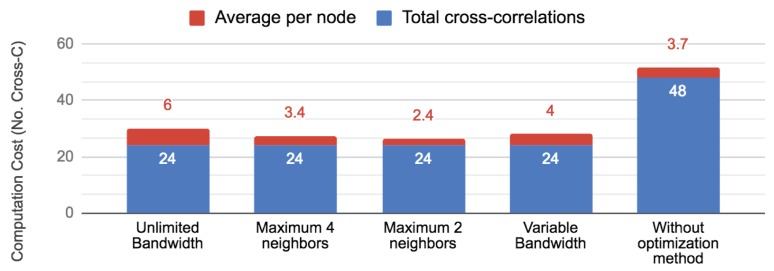
Computational cost in terms of number of cross-correlations computed by nodes. Comparison between our proposed communication-reduced method and communication pattern without reduction.Note that, in the case “unlimited bandwidth”, only 4/13 nodes computed cross-correlations, and, in the case “without optimization method”, all 13 nodes computed cross-correlations.

**Figure 15:**
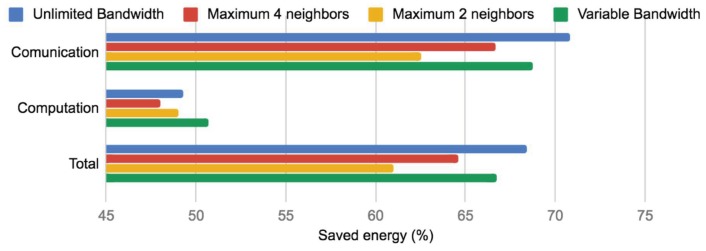
Percentages of energy saving respecting to the communication pattern without using out method.

The improvement in the communication cost is more than expected in this case; because of that, we analyze how our method impacts computation and energy saving. Figure 14 shows the computation cost of each experiment and the original communication pattern. Note that our method besides reducing communication cost also reduced computational cost because the cross-correlations were computed only once and at the “best nodes”. In this case, the experiment using a maximum of two neighbors was the one that better balanced the computation cost; however, the difference was not too significant respecting the other experiments that also used our method. Finally, we computed the percentage of energy saving when our method was applied. For doing this, our comparison was made respecting the original communication pattern. Figure 15 illustrates energy saving results. According to Pottie and Kaiser [28], the energy of transmitting 1 KB a distance of 100 m is approximately the same as executing 3 million instructions by a processor. Hence, local data processing and reducing communication cost is crucial for saving sensors energy. Note that, in terms of communication, in all our experiments, we saved at least more than 60% of energy. This is a very promising reduction. Furthermore, note that, in terms of computation, the energy saving in our experiments was around 50%. These results imply that our approach, besides reducing communication cost, also helped to avoid extra energy utilization.

Based on our evaluation, we remark that, when there is variable bandwidth that mainly relies on the number of links/hops of the nodes, our method performs very well in terms on communication reduction, computation reduction, and energy saving. Hence, we use the results of Experiment 4 to set up our system for a real-world deployment, as we explain in the next section.

## 5. Field Test and Evaluations

We deployed smart seismic sensor nodes at the University of Georgia to generate the velocity map and 3D structure of the subsurface using our dSPAC system and our optimized communication-reduced model. The previous systems demonstrated promising potential in illuminating either deep or shallow subsurface depending on the tomography method used within them [10,29,30]. However, one key problem of these systems is validation. For this reason, a validation test in a small/known area is suitable for validation purposes, especially in shallow ambient noise tomography. We allowws variable bandwidth based on the number of links/hops due to bandwidth and energy analysis presented in Section 4.5. In this section, we explain the used equipment, the deployment topology, the model used and the result of the real-world experiment. We show that our system could measure the velocity variations of the subsurface that corresponded with underground structures—in this case, a pipeline that is under our deployment location.

### 5.1. Smart Seismic Sensor Nodes

**Figure 16:**
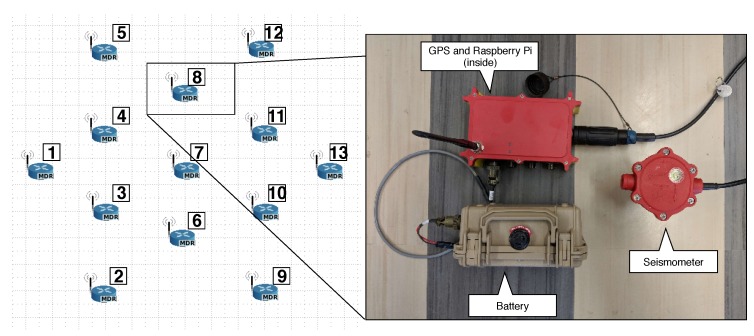
Deployment topology and smart seismic sensor nodes.

We used thirteen smart seismic sensor nodes (S3N) for a mesh network that communicates wirelessly. The instruments were placed in the field in a ring-based topology, as shown in Figure 16. Every S3N was composed of: (i) a Global Positioning System (GPS) that provided precise time-stamp and location information; (ii) a three-channel seismometer for getting vibration stream data; (iii) a computing board boxed into a waterproof box; and (iv) a waterproof battery 11 V and 99.9 Wh. Not that the computing board was basically a Raspberry Pi 3 with 1.2 GHz of CPU, 1 GB RAM and GPU for intensive local computing when needed, yet could be put in sleep for very low power consumption.

### 5.2. System Setup

dSPAC system was installed on each S3N. An automatic system service initiated the system process. The *data preparation* and *data selection* were automatically started and a mesh network was formed between nodes. Nodes were required to be synchronized. The synchronization was done via GPS. Once the node started, it obtained the timestamp from the GPS signal. Some nodes might receive the GPS signal before others and start to transmit; however, this transmission was discarded until all units were synchronized. Usually, this synchronization process did not take longer than 1 min. Once all nodes were synchronized via GPS, every node selected the best node to cross-correlate data and set this node to send the information. The cross-correlation window or cutting-time (λ) and the total time of the experiment (*T*) were set in a configuration file. The narrow frequency-band of interest was also indicated in this configuration file. This file was updated on all nodes by running a script before the process started. Every λ time, the data were processed and sent to the “best node(s)” for *cross-correlation*. After *T* time, the *subsurface imaging section* began by using the dSPAC coefficients to estimate the velocities. Every section of the system was developed in C++. An internal database in InfluxDB was used to store the data for future analysis if needed.

**Figure 17:**
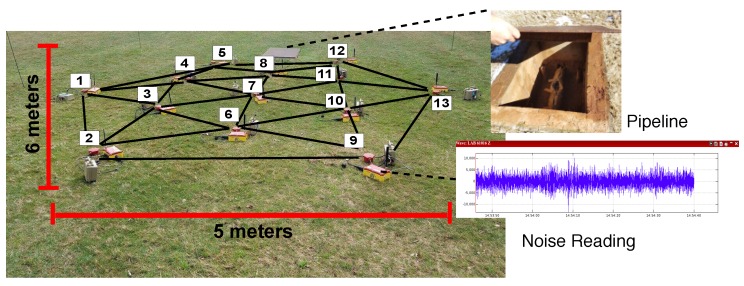
Deployment real location. A pipeline is located under the S3N location. Every node automatically started to read and cross-correlate the ambient noise. The distance between nodes was 3 m (9.843 ft.) from the center to the outside circle nodes, and 1.7 m (5.577 ft.) from the center to the inside circle nodes.

The real location of the nodes running the proposed system is shown in Figure 17.

### 5.3. Cross-Correlation Results

**Figure 18:**
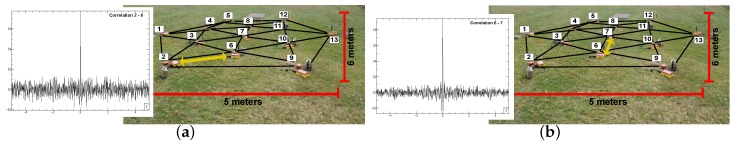
Cross-correlation results after stacking the measurements over 1 h: (**a**) $C_{2-6}$; and (**b**) $C_{6-7}$.

As mentioned, nodes sent the cross-correlation to their “best node” every λ time (in this experiment, λ was set in 5 min). The “best node” computed and stacksed the cross-correlation of its data with those from the neighbors. An example of cross-correlation between two nodes after 1 h of cross-correlation is shown in Figure 18.

Figure 18a is the cross-correlation between Node 2 and Node 6, which was calculated in Node 6. The cross-correlation in Figure 18b is between Nodes 6 and 7; this result was computed by Node 10.

### 5.4. Subsurface Imaging Results

After *T* hours of continuous system execution (in this experiment, *T* was 11 h, i.e., 660 min), the “best nodes” (nodes that computed the cross-correlations) returned the results of the stacked cross-correlation to the appropriate neighbors. Every node that was located in the center of each ring/sub-ring ran the dSPAC method, as explained in Section 2.3. Once the phase velocity estimation was completed for each sink node, they cooperatively constructed the velocity map for each depth layer. Then, the sensor located at the center of each ring used depth sensitivity kernel theory [31] to invert the frequency ranges in depth. It is worth noting that we could start sensing shapes after the first 5 min of correlations; however, due to the nature of ambient noise image, more data stacking improved the resolution. Then, more correlated data exhibited better performance (*T* = some hours). Our main advantage is the in-situ and real-time computing compared with other geophysical methods.

**Figure 19:**
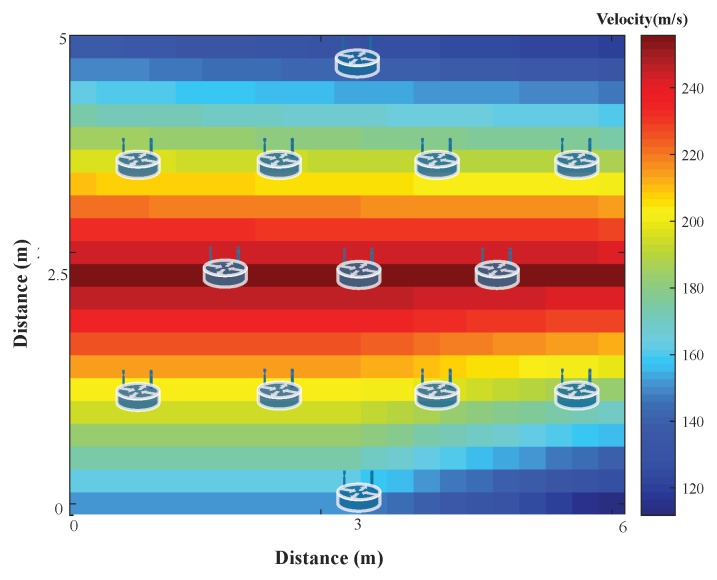
Velocity map of the layer ∼1.2 m depth. Sensor nodes locations are plotted as reference.

Figure 19 shows a layer of 1.2 m depth. The area with high velocity in the map indicated that it should be an isolated structure/facility, corresponding to the targeted pipeline. Notice that shallow depths had better resolution. Between 1 to 1.5 m, it was possible to distinguish a change in potential pipeline velocity. Depth layers showed changes in velocity but the resolution was low.

**Figure 20:**
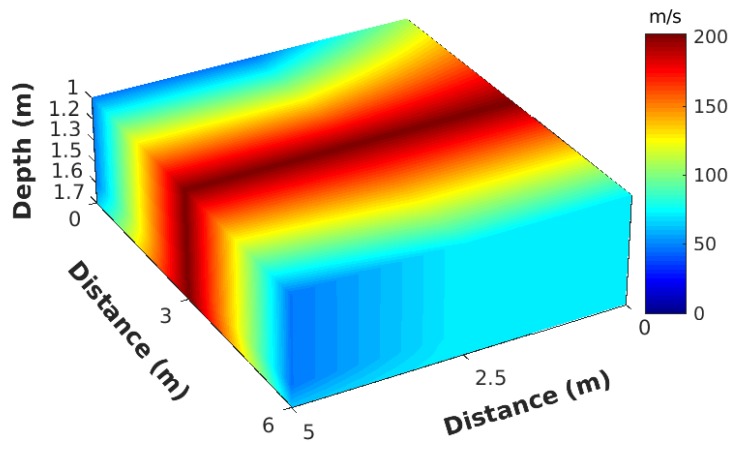
3D velocity subsurface: layers between 1 and 1.7 m.

The sink sensor constructed a 3D subsurface velocity image, as shown in Figure 20, by interpolating the velocity profiles from all the nodes. For instrument limitations, we chose 90 Hz as the dominant frequency in our experiments. The sampling rate of our sensors was 500 Hz. Based on the Nyquist–Shannon sampling theorem, only the first 250 Hz were usable. Furthermore, to avoid aliasing effect [32], we adopted up to 125 Hz frequencies. Because our goal was to only illuminate the shallow subsurface, we decided to use high frequencies of 80–100 Hz, which allows shallow velocities according to depth sensitivity kernel theory [31].

In Figure 20, only depths between 1 and 1.7 m are shown. In the center of the velocity map, we can notice the high-velocity area corresponding to the pipe location. Due to the high propagation velocity of the metal pipe, the surrounding soils also showed higher velocities than other areas. Horizontal resolution could be adjusted to a narrow frequency band, which was the most significant responses with the pipe to obtain a better resolution. Note that, as shown in Figure 19, in our application, the shallow subsurface velocity was around 200 m/s. Considering a central frequency of 90 Hz, the wavelength Λ (Λ=c/ω, where *c* is velocity and ω is frequency) was about 2.22 m/s. Then, the seismic resolution was calculated by Λ/4, resulting in our resolution being about 0.55 m, which is not optimal for a pipeline detection, whose diameter is about 20 cm. This is the reason the pipeline image looks thick in Figure 20. In addition, according to depth sensitivity kernel theory [31], the maximum depth for a frequency of 125 Hz is approximately 5–7 m, which differs at different locations with various geological conditions. Thus, the velocity map we generated is an average map between depth 0 and 5 m. Since the targeted pipe is located 1.3 m below ground, it should be detected in the imaging result. However, if the pipeline were not the only underground facility at this depth range, our result might be degraded. The solution to improve the resolution and image shallower subsurface was to increase the sampling frequency, which is the reason ground-penetrating radar (GPR) can do its job.

In addition, the vertical resolution could be further improved, if there were more stations. This result shows we could see structures under the subsurface and potentially extending our work for some security issues (for example, detecting broken pipelines, detecting tunnels, etc.).

## 6. Discussion about Robustness and Communication Limitations

In this section, we discuss: (i) the robustness of dSPAC approach and the recovery mechanism of the system when some nodes fail; and (ii) the communication limitations of the system and how it affects the communication and bandwidth analysis.

### 6.1. Robustness of the System

Any distributed system needs to overcome problems in the scenario of sensor failures. Suppose that, during time *T*, one or more nodes fail, and suppose that these nodes are not the nodes that compute cross-correlations. Every S3N has been designed to restart automatically the operations after failure. However, during the time the node is down, the other nodes continue working sending data for cross-correlation to the “best node”. At the moment the node is automatically restarted, it synchronizes itself via GPS with the rest of the nodes, and it continues the cross-correlation of the data from that point. Because, after cross-correlation, the system stacks the results (*time-stacking*), the short-time failure does not affect the reliability of the cross-correlations. This guarantees self-healing and resilience of the system. Now, suppose that during time *T* one of the “best nodes” fails. Similarly, the node restarts automatically and re-synchronizes via GPS. Because every S3N is equipped with an internal database, the cross-correlation calculation before the failure is saved, and the stacking process can continue after recovery. Some data in the middle are lost, but, once again, due to the stacking process, this small lost does not affect the system if the time of failure is short.

**Figure 21:**
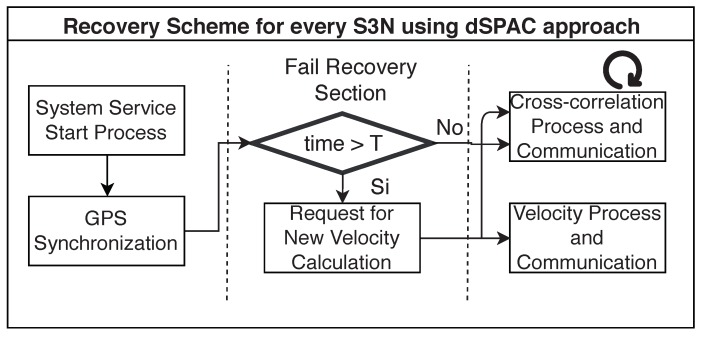
Recovery scheme for system resilience after failures from the sink nodes perspective.

However, for the “subsurface imaging” process, the loss of one of the sink sensors is crucial for the velocity assembling and interpolation. For this reason, we designed a recovery scheme for recalculating the velocity map after a sink sensor failure. The scheme is described in Figure 21 from a sink sensor perspective.

In this scheme, after a sensor is automatically started with a system service, and it has been synchronized with the other sensors, the sensor checks if there is a velocity calculation and imaging has been done during the time it was down. This is done by checking whether the current time is greater than the time the process supposes to be performed. If this happens, the sink node sends a request to other sink nodes for recalculation of the velocities and interpolation. The cross-correlation process also starts in any case. Note that, to avoid energy failures, we can introduce solar panels for recharging batteries. With this scheme, we introduce resilience to the system, and we aim to guarantee that the results will be computed with the maximum number of available sink sensors.

### 6.2. Communication Limitation Discussion

In the presented methodology, neighbor communication is fundamental to avoid centralized approaches. However, it is important to discuss the effect of the communication range in the potential applications of dSPAC deployments and methodology.

In a mesh network, every “*hop*” (link) between sensors will decrease the bandwidth by half [26]. This happens because wireless links can only do one thing at a time—transmit or receive. In a long “chain” of mesh links, this results in a very slow connection from end to end. If all nodes are required to transmit to a central place, the nodes near to the central node have less bandwidth, and some of them would become a bottleneck. For this reason, we are proposing the communication only between neighbors. However, what is the maximum distance between two nodes to actually communicate without interruptions and collisions? It depends on the communication type. If we use a wireless network, as we propose here, the distance between nodes can be 20–50 m. If we use XBee communication, it may allow a communication range up to 45 km with a high gain antenna [33], which means that our approach can be applied to bigger deployments.

To illustrate the advantage of having neighborhood communication instead centralized communication, we present two studies: (i) a comparison of throughput using neighbor communication (distributed) vs. centralized communication; and (ii) a comparison of communication cost between the same two scenarios

We calculated the available bandwidth based on our hardware limitations and the throughput of the network at each time point. Then, we compared the distributed approach proposed in this paper, with the centralized approach. Our instruments are based on a Raspberry Pi 3 as computer board (computational unit inside S3N). The wireless communication bandwidth of Raspberry Pi 3 is estimated at ∼10 Mbps (Megabytes per second) [27]. Due to the number of links in our topology (some nodes may have five or six links, which reduced the available bandwidth), we based our observations on a maximum available bandwidth of ∼2 Mbps.

**Figure 22:**
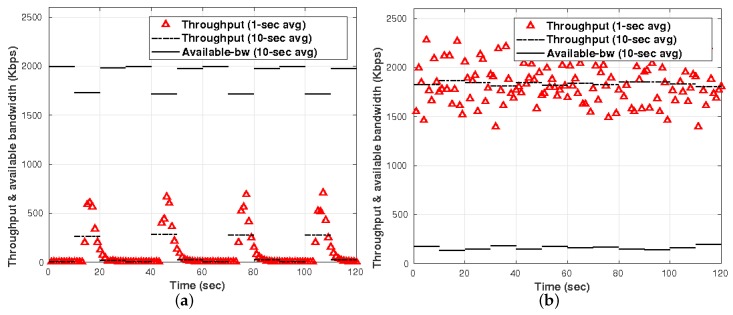
Throughput and bandwidth availability in: (**a**) distributed approach; and (**b**) centralized approach.

Figure 22 shows the comparison between the distributed and centralized approaches. This throughput was recorded for 120 s in which nodes in the distributed approach exchange information with the neighbors every *t* = 20 s to perform cross-correlation later. In the centralized approach, the nodes are all the time sending raw data to the central place, and we can notice that the average available bandwidth is very low all the time. On the other hand, with our distributed approach, the available bandwidth only has a small decrease during transmission for cross-correlation. Our approach meets the bandwidth limitations, and the sent packages are small due to data preparation and compression.

The system performance based on communication cost was also analyzed for the proposed approach. Because the most intensive communication scenario occurs when the data are continuously transmitted for cross-correlation, we present the communication cost after 1 h of transmission.

**Figure 23:**
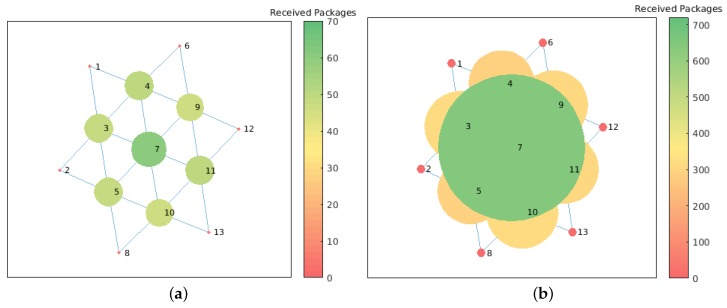
Communication cost in terms of number of received messages by each node. Communication of data for cross-correlation after 1 h of execution: (**a**) distributed approach—number of messages between 0 and 70; and (**b**) centralized approach—number of messages between 0 and 700.

In Figure 23a,b, we can see that communication cost in a centralized setup is high near the “central node” as all the raw data are transferred over the network. It is worth noting that the distributed approach improves significantly the communication cost between nodes. The reduction in the number of received messages is ∼75%. This also has an impact in the energy consumption of each node. According to to Pottie and Kaiser [28], the energy of transmitting 1 KB a distance of 100 m is approximately the same as executing three million of instructions by a processor. Hence, local data processing is also crucial for saving sensors energy. This implies that our approach, besides reducing communication cost, also helps avoid extra energy utilization.

## 7. Future Work

With the obtained results, we envision our methodology and system can be applied to other applications for shallow subsurface imaging. For example, we aim to utilize our method to detect water leakage in shallow-buried pipelines. Since water saturation may affect the underground velocity, we believe our method may be suitable for this application. Similarly, near surface seismic imaging helps monitor shallow buried objects [9,34,35], for example, very shallow seismic reflection and refraction experiments can be conducted to investigate groundwater level changes in beach sand in situ [36]. These are other potential applications that we aim to explore with our methodology.

## 8. Conclusions

In this paper, we present a communication-reduced method for cross-correlation of ambient noise data for subsurface imaging using distributed spatial auto-correlation (dSPAC). The main idea is to reduce the communication cost between nodes when they are working together to correlate data. The subsurface methodology includes two main sections: cross-correlation section and subsurface imaging section. The main contribution of this paper is based on the cross-correlation section. We have shown that is possible to select “best nodes” to correlate the needed data for imaging, and, at the same time, meet bandwidth constraints. We also present analysis regarding computational cost and energy consumption of the nodes. We integrated our optimal solution to a real-world deployment, and we imaged subsurface structures that are close to the ground truth. The potential scientific and social impact of our method is significantly and broadly widespread.

## Figures and Tables

**Table 1:** Communication model variables.

Variable	Description
$\lambda_{uv}$	Communication cost between nodes *u* and *v*.
$\beta_{uv}$	Number of Packets between nodes *u* and *v*.
$\chi_{u}$	Maximum number of packets to transmit simultaneously by a node *u*. ($\chi_{u}=\Omega_{u}/S_{u}$).
$\Omega_{u}$	Available bandwidth.
$S_{u}$	Size of the packet to be sent by *u*.
*h*	Gain for each node connection between Layer 2 and Layer 3 in $G^{\prime }$.
$\Gamma_{u}$	Set of nodes connected by output edges with node *u* Outflow.
$\Upsilon_{u}$	Set of nodes connected by input edges with node *u* Inflow.
*r*	Set of intermediate nodes with gain.
*g*	set of intermediate nodes without gain.

**Table 2:** Needed cross-correlations for the Figure 7 topology.

Needed Cross-Correlations					
$C_{1-3}$	$C_{3-4}$	$C_{4-7}$	$C_{6-9}$	$C_{7-11}$	$C_{10-11}$
$C_{1-4}$	$C_{3-6}$	$C_{4-8}$	$C_{6-10}$	$C_{8-11}$	$C_{10-13}$
$C_{2-3}$	$C_{3-7}$	$C_{5-8}$	$C_{7-8}$	$C_{8-12}$	$C_{11-12}$
$C_{2-6}$	$C_{4-5}$	$C_{6-7}$	$C_{7-10}$	$C_{9-10}$	$C_{11-13}$

**Table 3:** Number of cross-correlations for each selected node (unlimited bandwidth).

Node Number	Computed Cross-Correlation	Node Number	Computed Cross-Correlation
2	$C_{2-3}$	10	$C_{6-7}$
	$C_{2-6}$		$C_{6-9}$
	$C_{3-6}$		$C_{6-10}$
4	$C_{1-3}$		$C_{7-10}$
	$C_{1-4}$		$C_{7-11}$
	$C_{3-4}$		$C_{9-10}$
	$C_{3-7}$		$C_{10-11}$
	$C_{4-5}$		$C_{10-13}$
	$C_{4-7}$		$C_{11-13}$
	$C_{4-8}$	11	$C_{8-11}$
	$C_{5-8}$		$C_{8-12}$
	$C_{7-8}$		$C_{11-12}$

**Table 4:** Number of cross-correlations for each selected node (limited bandwidth up to 4 neighbors).

Node Number	Computed Cross-Correlation	Node Number	Computed Cross-Correlation
4	$C_{1-3}$	3	$C_{2-3}$
	$C_{1-4}$		$C_{2-6}$
	$C_{3-4}$		$C_{3-6}$
	$C_{3-7}$	7	$C_{7-8}$
	$C_{4-7}$	10	$C_{6-9}$
6	$C_{6-7}$		$C_{6-10}$
8	$C_{4-5}$		$C_{9-10}$
	$C_{4-8}$	11	$C_{7-10}$
	$C_{5-8}$		$C_{7-11}$
	$C_{8-11}$		$C_{10-11}$
	$C_{8-12}$		$C_{10-13}$
	$C_{11-12}$		$C_{11-13}$

**Table 5:** Number of cross-correlations for each selected node (limited bandwidth up to 2 neighbors).

Node Number	Computed Cross-Correlation	Node Number	Computed Cross-Correlation
1	$C_{1-3}$	6	$C_{6-9}$
	$C_{1-4}$		$C_{6-10}$
	$C_{3-4}$		$C_{9-10}$
2	$C_{2-3}$	8	$C_{7-8}$
	$C_{2-6}$	10	$C_{7-10}$
	$C_{3-6}$		$C_{7-11}$
3	$C_{3-7}$	11	$C_{8-11}$
4	$C_{4-5}$		$C_{8-12}$
	$C_{4-7}$		$C_{11-12}$
	$C_{4-8}$	13	$C_{10-11}$
	$C_{5-8}$		$C_{10-13}$
7	$C_{6-7}$		$C_{11-13}$

**Table 6:** Number of cross-correlations for each selected node (variable bandwidth).

Node Number	Computed Cross-Correlation	Node Number	Computed Cross-Correlation
3	$C_{1-3}$	8	$C_{7-8}$
	$C_{1-4}$		$C_{7-11}$
	$C_{3-4}$		$C_{8-11}$
	$C_{3-7}$		$C_{8-12}$
	$C_{4-7}$		$C_{11-12}$
4	$C_{4-5}$	10	$C_{6-7}$
	$C_{4-8}$		$C_{6-9}$
	$C_{5-8}$		$C_{6-10}$
6	$C_{2-3}$		$C_{7-10}$
	$C_{2-6}$		$C_{9-10}$
	$C_{3-6}$	13	$C_{10-11}$
			$C_{10-13}$
			$C_{11-13}$
